# Change of government’s subsidization policy improves smoking cessation services: a cross-sectional study from the perspectives of physicians

**DOI:** 10.1186/s12889-016-3052-7

**Published:** 2016-05-17

**Authors:** Tai-Yin Wu, Ling-Yu Hung, Wei-Chu Chie, Tai-Yuan Chiu, Fei-Ran Guo

**Affiliations:** Department of Preventive Medicine, Renai Branch, Taipei City Hospital, Taipei, Taiwan; Institute of Epidemiology and Preventive Medicine, College of Public Health, National Taiwan University, Taipei, Taiwan; Department of Family Medicine, Yonghe Cardinal Tien Hospital, New Taipei City, Taiwan; Department of Family Medicine, National Taiwan University Hospital & College of Medicine, Taipei, Taiwan

**Keywords:** Government’s subsidization, Smoking cessation services, Cross-sectional study

## Abstract

**Background:**

The Taiwanese government increased financial subsidies for smoking cessation services in 2012. We aimed to evaluate the effects of this new policy on smoking cessation services from the physician’s perspective.

**Methods:**

This was a cross-sectional nationwide survey. Physicians who provided smoking cessation services for more than ten patient encounters in the preceding year of the new policy (February 2011 to March 2012) were recruited. The questionnaire was developed by two experts and was validated by a committee consisting of 11 delegates.

**Results:**

We sent a total of 1,319 questionnaires. The response rate was 45.9 %. The majority of respondents were male (88.4 %), middle-aged (65.3 %), and worked as family physicians (56.1 %). Most physicians agreed that the new policy had increased the number of patients seeking smoking cessation, increased patients’ willingness to adopt pharmacotherapy, helped physicians to prescribe medications, improved patients’ adherence to medications, and improved quality of care. These changes were most prominent in medical centers. Changes in the practice of the 5As (ask, advise, assess, assist, arrange) were moderate. Among different medical settings, the most significant change was an increase in the expenditure on smoking cessation medications.

**Conclusions:**

The new subsidization policy in Taiwan has improved smoking cessation services. Overall, physicians reported positive effects of the new policy. Further study is warranted to evaluate the long-term influence of the policy.

## Background

Tobacco use has reached epidemic levels around the world, resulting in a worldwide increase in tobacco-related deaths and disabilities [1]. In 2012, smoking rates for men and women in Taiwan were 32.7 and 4.3 %, respectively [2]. Wen and colleagues report that smoking-related diseases are responsible for a loss of 22 years’ life expectancy in Taiwanese smokers [3].

There is strong evidence to support a need for tobacco control programs to decrease the health and economic burden of smoking [4]. An important strategy is to offer help to quit tobacco use [5]. Article 14 of the World Health Organization Framework Convention on Tobacco Control (WHO FCTC) states that national policy or health insurance systems should make medications accessible and free, or at an affordable cost [6]. In the International Tobacco Control 4 Country Survey, under national financial policy (English Stop Smoking Services), U.K. smokers were more likely to achieve 28-day abstinence than those in Australia, Canada, and the United States [7].

The Taiwanese government has taken multiple approaches to reduce tobacco demand in different phases [8]. Since 2002, a tobacco health and welfare surcharge of NT$5 (US$0.14) per pack of cigarettes has been imposed [9], and subsidized tobacco cessation services that provide pharmacotherapy and brief counseling by physicians have been initiated [10]. Following the amendment of the Tobacco Hazards Prevention Act in 2009, smoking has been prohibited in virtually all indoor areas [11]. Over the years, the specialties providing smoking cessation services have evolved from family physicians and internists only to all medical specialties, including pediatrics, obstetrics, and psychiatry. Many Taiwanese physicians have been trained to help patients quit smoking, and smoking cessation has become a popular service [12]. The findings of the 2012 Adult Smoking Behavior Survey in Taiwan revealed that half of the responding smokers (50.7 %) had been urged by medical professionals to quit smoking [2]. The abstinence rates of the outpatients in one medical center were 99.7, 49.2, 37.7, 30.2, and 22.7 %, at 1, 3, 6, 12, and 36 months, respectively [9].

By 2012, smoking cessation services were available at 2,152 contracted medical institutions with a geographic coverage rate of 97 % [2]. The physician fee was paid by the Health Promotion Administration (HPA) at a rate of NT$250 (US$8.33) per visit. Patients were responsible for a weekly out-of-pocket prescription co-payment of NT$550 to NT$1,250 (US$18.33 to US$21.67) [2]. However, this cost was still a potential hurdle for people on low incomes who intended to quit smoking.

To remove the economic barrier of smoking cessation service and lessen health inequality, the government started the Second-generation Cessation Payment Scheme (SCPS) in March 2012. The new policy is as follows: the patient’s co-payment is 20 % of the total medication cost and has an upper limit of NT$200 (US$6.67). The upper limit allows physicians to prescribe more expensive medications such as varenicline without being concerned about cost. Full subsidies are provided for smokers from low-income households. To encourage tracking of the smoking status of the patients, case management fees are also subsidized at NT$100 (U$3.33) per treatment course. Case managers have to call patients by telephone and report 7-day point prevalence rates at 3- and 6-months. The new policy also includes some other changes. The maximum length of prescription is extended from 2 to 4 weeks, and cessation services are no longer restricted to outpatients but also serve inpatients and emergency room patients.

After SCPS, the number of smoking cessation patients increased by 41 %, from 83,724 during the year before its implementation to 117,989 during the year after implementation [13]. The 7-day point prevalence rate at 6 months increased from 22.8 to 28.0 % [14]. However, we don’t know the extent of these changes, such as the patient’s willingness to adopt pharmacotherapy, patient’s adherence to medication, and the quality of care. We also don’t know the attitude of the physicians (positive or negative to the new policy), the barriers of implementation, and the change of the physicians’ practice. In this study, we aimed to evaluate the impact of SCPS from the perspectives of physicians.

## Methods

### Study design

We conducted a cross-sectional nationwide questionnaire-based survey.

### The participants

We recruited 1,319 physicians who provided smoking cessation services for more than ten patient encounters in the preceding year of the new policy (February 2011 to March 2012). The physicians’ roster was provided by the HPA, Ministry of Health and Welfare, Taiwan.

### The questionnaire

The questionnaire was designed by two family physicians specializing in smoking cessation (FR Guo and LY Hung). An expert committee composed of 11 delegates from the Taiwan Association of Family Medicine, Taiwan Society of Internal Medicine, Taiwanese Society of Psychiatry, and HPA then collectively reviewed the questionnaire. A five-point Likert scale was used to encode the content validity of the questionnaire according to the importance and appropriateness of each item. The criterion of an average score of four or above was used to select the final items.

The questionnaire included five domains: patient changes, physicians’ attitudes towards the new policy, barriers to implementation, changes in practicing the 5As (ask, advise, assess, assist, arrange), and institutional changes. Each domain was composed of five simple choice questions. Except for the domain of institutional changes, which were yes/no questions, the other four domains were five-point Likert scale choices. For these Likert-scaled questions, the choices were recorded as scores. A score of one meant strong disagreement while five meant strong agreement. The average score reflected the level of physicians’ agreement with the statement. By combining the scores of the five questions in each domain, we obtained a summation index of each domain (including patient changes, physician attitudes, possible barriers, and practice of the 5As).

### The process of sending the questionnaires and collecting responses

We mailed the questionnaires to each physician’s practicing institution in July 2012. Physicians had to sign an informed consent before study participation. To improve the response rate, participants could respond in one of three ways: in a pre-paid return envelope, by facsimile, or by e-mail. We examined the questionnaires 2 weeks later and resent the same questionnaire in August to non-respondents. After collecting the last questionnaires on September 20, 2012, we started data analysis. Questionnaires without a physician’s signature were excluded. A questionnaire was considered “valid” if it had less than three unanswered questions. A small gift of appreciation, a LED lamp, was sent to respondents who provided valid questionnaires.

### Statistical analysis

Descriptive analysis was used for baseline characteristics and variables in the questionnaire. We examined the difference between respondents and non-respondents using a chi-square test. For multivariate analysis, we used baseline characteristics as independent variables and summation indexes as dependent variables. The correlations were examined by multiple regression models. To evaluate the institutional changes in different levels of medical settings, we further stratified our data according to settings, i.e., medical centers, regional hospitals, local hospitals, and primary care facilities. Chi-square tests were used to examine the differences. A p-value <0.05 for two-tailed tests was considered statistically significant. SPSS for Windows (IBM Company, New York, USA) was used for statistical analysis.

### Ethical approval

This study was approved by the institutional review board (IRB) of Cardinal Tien Hospital, Taiwan. Only physicians who signed the informed consents were included in the analysis. Taiwan Association of Family Medicine is responsible for the certification of physicians and the researchers of this study have accessed the certification records. The researchers did not access patient records.

## Results

We sent a total of 1,319 questionnaires. The response rate was 45.9 % (*n* = 605). The majority of the questionnaires were received by mail (*n* = 429, 70.9 %), followed by fax (*n* = 162, 26.8 %), and e-mail (*n* = 14, 2.3 %). Compared to non-respondents, respondents tended to be older, mostly family physicians, and mainly practicing in primary care settings (Table 1). The majority of respondents practiced in private institutions (*n* = 465, 76.9 %).

**Table 1:** Baseline characteristics of the respondents and non-respondents

Characteristics	Respondents (*n* =605)		Non-respondents (*n* =714)		*p*
	n	(%)	n	(%)	
Gender					
Male	535	(88.4)	628	(88.0)	0.79
Female	70	(11.6)	86	(12.0)	
Age (years)					
39 and under	103	(17.0)	162	(22.7)	0.001
40-59	395	(65.3)	470	(65.8)	
60 and over	107	(17.7)	82	(11.5)	
Specialty					
Family medicine	339	(56.1)	324	(47.7)	0.009
Internal medicine	142	(23.5)	166	(24.4)	
Psychiatry	10	(1.7)	17	(2.5)	
Others	114	(18.8)	172	(25.3)	
Practicing institution					
Medical center	25	(4.1)	40	(5.6)	<0.001
Regional hospital	61	(10.1)	190	(26.7)	
Community hospital	56	(9.3)	11	(1.5)	
Primary care setting	463	(76.5)	471	(66.2)	
Smoking status					
Non-smoker	531	(87.8)	-	-	
Ex-smoker	68	(11.2)	-	-	
Current smoker	6	(1.0)	-	-	

Table 2 presents the impact of the new subsidization policy to smoking cessation services. Most physicians agreed or strongly agreed that the new policy increased the number of patients engaging in smoking cessation treatment (85.3 %), and increased patients’ willingness to adopt pharmacotherapy (55.0 %). For physicians’ attitudes, more than 50 % of respondents agreed or strongly agreed with all five items, including that the new policy was an improvement over the previous policy. The quality of care was also deemed to have improved. For possible barriers to the implementation of the new policy, the majority of physicians were neutral, and less than 50 % agreed or strongly agreed with all five items. It was most difficult to track the abstinence rates of patients (39.8 %) and least difficult to use the information system (24.0 %). Regarding the practice of the 5As, changes following the introduction of the new policy were moderate. The percentages of physicians who agreed or strongly agreed that the change in practice of the 5As was improved ranged from 44.2 % (for arrange) to 48.7 % (for assist), respectively.

**Table 2:** The impact of the new subsidization policy to smoking cessation services

Impact	Strongly disagree & disagree		Neutral		Agree & strongly agree	
	*n*	(%)	*n*	(%)	*n*	(%)
Patient changes						
Increase in the number of smoking cessation patients	58	(9.6)	31	(5.1)	516	(85.3)
Increase in follow-up visits of smoking cessation patients	206	(34.0)	147	(24.3)	252	(41.6)
Increase in patients’ willingness to adopt pharmacotherapy for smoking cessation	145	(24.0)	127	(21.0)	333	(55.0)
Increase in the number of smoking cessation patients referred by other patients	173	(28.6)	175	(28.9)	256	(42.4)
Increase in patients’ abstinence rate	129	(21.3)	176	(29.1)	299	(49.6)
Physician’s attitudes towards the new policy						
The new policy encourages more patients to quit smoking	113	(18.7)	130	(21.5)	362	(59.8)
The new policy helps me to prescribe smoking cessation medications	71	(11.7)	112	(18.5)	422	(69.8)
The new policy improves patients’ adherence to medications	116	(19.2)	173	(28.6)	315	(52.1)
The new policy improves quality of care	87	(14.4)	176	(29.1)	341	(56.5)
Overall, the new policy is an improvement over the previous policy	87	(14.4)	143	(23.6)	375	(61.9)
Possible barriers to the implementation of the new policy						
Difficulties in providing services to inpatients and emergency room patients	77	(12.7)	304	(50.2)	222	(36.8)
Difficulties in tracking the abstinence rate of the patients	161	(26.6)	203	(33.6)	240	(39.8)
Difficulties in reimbursement and case report processes	162	(26.8)	270	(44.6)	172	(28.4)
Difficulties in applying for case management fees	138	(22.8)	265	(43.8)	201	(33.2)
Difficulties in using the information system	182	(30.1)	277	(45.8)	145	(24.0)
Changes in practicing 5As after the new policy						
Increase in asking patients’ tobacco use status	85	(14.1)	245	(40.5)	274	(45.4)
Increase in advising patients to quit smoking	81	(13.4)	230	(38.0)	293	(48.5)
Increase in assessing patients’ willingness to quit	88	(14.5)	225	(37.2)	291	(48.2)
Increase in assisting patients to quit smoking	116	(19.2)	194	(32.1)	294	(48.7)
Increase in arranging follow-up visits	97	(16.0)	240	(39.7)	267	(44.2)

We used multiple regression models to evaluate the correlation between baseline characteristics and four summation indexes (Table 3). Medical centers were the institution type most associated with patient changes, physician attitudes and practice of the 5As. Age and the specialty of family medicine were also associated with patient changes. The former had an inverse association while the latter had a positive association. No other variable of baseline characteristics was significantly associated with the summation indexes.

**Table 3:** Multiple regression models of summation indexes

Factor	Patient changes		Physician attitudes		Possible barriers		Practice ^a^5As	
	ß	*p*	ß	*p*	ß	*p*	ß	*p*
Physician’s age	−0.03	0.045	−0.02	0.25	−0.01	0.89	−0.01	0.55
Physician’s gender: male	0.05	0.93	0.16	0.77	−0.23	0.55	0.88	0.06
Specialty: family medicine	0.68	0.03	0.57	0.09	0.19	0.42	0.35	0.22
Institution: medical center	2.94	0.001	3.36	0.001	1.13	0.06	2.34	0.001
Smoking status: non-smoker	0.07	0.89	0.45	0.37	−0.27	0.46	0.44	0.32

^a^5As: ask, advice, assess, assist, arrange

Table 4 presents institutional changes at different levels of medical setting. The most prominent change observed was an increase in expenditure on smoking cessation medications (52.9 %). This change was consistent across different levels of settings. Only a few institutions reported an increase in smoking cessation personnel; however, medical centers had significantly greater increases in the numbers of physicians, case managers, and professional counselors providing smoking cessation services.

**Table 4:** The impact of new subsidization policy to institutional changes in different levels of medical settings

Impact	Total (*n* = 605)		Primary care settings (*n* = 463)		Community hospitals (*n* = 56)		Regional hospitals (*n* = 61)		Medical centers (*n* = 25)		*p*
	*n*	(%)	*n*	(%)	*n*	(%)	*n*	(%)	*n*	(%)	
Increase in smoking cessation clinics	167	(27.6)	125	(27.0)	15	(26.8)	15	(24.6)	12	(48.0)	0.134
Increase in physicians providing smoking cessation services	113	(18.7)	66	(14.3)	16	(28.6)	14	(23.0)	17	(68.0)	<0.001
Increase in expense of smoking cessation medications	320	(52.9)	249	(53.8)	25	(44.6)	31	(50.8)	15	(60.0)	0.515
Increase in case managers in charge of the follow-up of patients	97	(16.1)	58	(12.6)	14	(25.0)	15	(24.6)	10	(40.0)	<0.001
Increase in professional counselors	76	(12.6)	42	(9.1)	10	(17.9)	13	(21.3)	11	(44.0)	<0.001

Comparing physicians’ responses from different medical settings (data not shown), physicians at medical centers seemed to take a more positive attitude towards the new policy. However, they also encountered more difficulties in providing services to inpatients and emergency room patients.

For the reliability of the questionnaire, the Cronbach’s Alpha of the domain of patient changes is 0.81, physician’s attitudes is 0.92, barriers to implementation is 0.69, and changes in practicing 5As is 0.88, respectively.

## Discussion

We observed that, in half a year, physicians reported that the new subsidization policy in Taiwan resulted in an increased number of patients, better prescription of medications, improved patient adherence, and improved quality of care. These changes were in accordance with the reduced medication cost to the patients. The impact was more prominent in medical centers, and being a family physicians was also associated with overall ratings in patient changes.

There is substantial evidence for the efficacy of financial incentives for smoking cessation. In a Cochrane review, financial interventions had a favorable effect on abstinence at 6 months (Relative risk, RR: 2.45, 95 % confidence interval, CI 1.17 to 5.12), the number of participants making an attempt to quit (RR: 1.11, 95 % CI 1.04 to 1.32) and the use of smoking cessation treatment (RR: 1.83, 95 % CI 1.55 to 2.15) [15]. The incentive of reimbursement was the most significant factor affecting physicians’ confidence and adherence to practice guidelines [12]. In Taiwan, the cost barrier was partially removed in 2002, when the Taiwanese government became the second in the world to reimburse smoking cessation, just behind England. In a cost-benefit analysis evaluating the Taiwanese smoking cessation services, the benefits far exceeded the costs after accounting for the different abstinence, relapse, and discount rates [16].

We proposed that if more than half (>50 %) of the responding physicians agreed or strongly agreed with a certain item, then such change was meaningful. It is worth noting that 52.9 % of the physicians agreed that the expenditure on smoking cessation medications increased after the introduction of the new policy. Varenicline, a relatively new pharmacotherapy for smoking cessation [17], confers a higher abstinence rate of up to three years [18]. Varenicline is also more costly. Sueh et al. report that among U.S. Medicare beneficiaries, greater varenicline out-of-pocket expense is significantly associated with lower adherence and lower likelihood of receiving a refill (odds ratio, OR: 0.59, 95 % CI 0.54 to 0.66) [17]. Under the SCPS in Taiwan, physicians have more freedom to prescribe appropriate medication according to individual needs rather than being restricted by pharmaceutical prices. The prescription of varenicline increased dramatically after the introduction of SCPS. It is suggested that the increased abstinence rate may be associated with the increased use of varenicline [14]. In a way, the new policy has achieved its main goal: to remove the economic inequality.

The new policy has resulted in rises in the number of clinical visits and patients using smoking cessation services. More than four-fifths of physicians agreed or strongly agreed that the most striking change brought about by the new policy was an increase in patients. Case numbers increased by 41 % after the implementation of the new policy [13].

Although the new policy has moderately changed the practice of the 5As, physicians in medical centers took a more positive attitude towards the new policy. This is probably because medical centers are more apt to allocate human resources to smoking cessation services, including physicians, case managers, and professional counselors. Medical centers also experienced greater changes with respect to the domains of patient changes, physicians’ attitudes and practice of the 5As. The medical centers in Taiwan are different from those in the western countries. A medical center not only provides intensive care, cancer treatment and specialty consultations, it also provides large volume of outpatient services. A patient may walk in a medical center without referral. Therefore, the medical centers in Taiwan may provide more accessibility to smoking cessation services as compared to those in many western countries.

The majority of the physicians were neutral about possible barriers to the new policy, probably because the smoking cessation service has been in operation since 2002. Physicians are already familiar with the computerized information system and have little difficulties in using it. Physicians in hospitals experience more difficulties in providing services to inpatients and emergency room patients. Though the new policy extends services to these two patient groups, there are greater barriers of implementation.

We recruited physicians who might have higher levels of performance and self-efficacy in assisting patients to quit smoking. Physicians with better confidence might be more compliant with practice guidelines [19]. Just like previous surveys, family physicians were more likely to respond than internists [12], probably due to more training in counseling skills [20]. Family physicians also experienced more favorable patient changes. Younger physicians who have undergone newer training programs might be more familiar with the issue of smoking cessation. Ulbricht S et al. reported younger general practitioners provided more smoking cessation counseling [21]. In our study, younger physicians reported more patient changes compared to their older peers.

This study has certain strengths. We obtained a nationwide representative list of physicians providing smoking cessation services. Our study might more accurately reflect the true picture of daily practice in different medical settings than previous studies. To increase response rate, we allowed for different ways of responding. It is well recognized that Taiwanese physicians are reluctant to answer questionnaires [12]. Compared to similar surveys targeting physicians providing smoking cessation services, which had a 38 % response rate in 2007 or 6.7 % in 2011 (unpublished data), this study had the highest response rate (45.9 %) among the studies carried out by the Taiwan Association of Family Medicine. We evaluated service changes as well as changes at different institutional levels. As far as we know, this is the first study to compare smoking cessation attributes across different medical settings.

Certain limitations existed, such as the presence of selection bias. Our participants might be more in favor of the new policy compared to non-respondents. There were differences in baseline characteristics between respondents and non-respondents, and the study’s conclusions might not be generalizable to the whole population. We were not able to achieve long-term follow up, particularly with respect to the decline in smoking rates. We enrolled only physicians providing smoking cessation services, who tend to be more supportive of policies that favor such practices. The attitude of physicians not enrolled in smoking cessation services remains unknown. The Chinese culture makes people tend to choose the “right” answer rather than the objective truth, therefore, we might have overestimated the positive effects of the new policy.

Future studies may include physicians who are less engaged in smoking cessation services, but play important roles in tobacco control, such as pulmonologists and cardiologists. Future studies may also focus on cost-benefit analysis, or the influence of more expensive treatments such as varenicline and combination nicotine replacement therapy. Since this study was carried out shortly after the implementation of the new policy, it is not known whether the attitudes of physicians would change over time. Potential changes in physician attitudes could be examined in future longitudinal studies.

## Conclusions

We observed that the Second-generation Cessation Payment Scheme in Taiwan improved smoking cessation services. Overall, physicians agreed that the effects of the new policy were positive. The enactment of the new reimbursement policy seems to have attained its goal of promoting smoking cessation in the short term. Subsequent follow up is mandatory to evaluate the long-term effects.

### Availability of data and materials

The dataset supporting the conclusions of this article is available in the Open Science Framework repository at https://osf.io/dkvtw/.

## Abbreviations

5As: ask, advise, assess, assist, arrange
CI: confidence interval
HPA: Health Promotion Administration
NT$: new Taiwan dollar
OR: odds ratio
RR: relative risk
SCPS: second-generation cessation payment scheme
WHO FCTC: World Health Organization Framework Convention on Tobacco Control
